# The Multipurpose Application WeChat: A Review on Recent Research

**DOI:** 10.3389/fpsyg.2018.02247

**Published:** 2018-12-11

**Authors:** Christian Montag, Benjamin Becker, Chunmei Gan

**Affiliations:** ^1^Molecular Psychology, Institute of Psychology and Education, Ulm University, Ulm, Germany; ^2^neuSCAN Laboratory, The Clinical Hospital of Chengdu Brain Science Institute, MOE Key Laboratory for Neuroinformation, University of Electronic Science and Technology of China, Chengdu, China; ^3^School of Information Management, Sun Yat-sen University, Guangzhou, China

**Keywords:** WeChat, WeChat addiction, motivation, uses and gratification, personality, Facebook, WhatsApp, social media

## Abstract

With currently over one billion monthly active users, the Chinese social media and multipurpose application WeChat (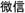, *Wēixìn*, micro-message) has become one of the world’s most popular social media platforms. Despite its enormous number of users in Asia, WeChat is still not well known in Western countries. Against this background, the present review aims to provide the reader with a comprehensive overview on the functionality of this application, comparison with other popular applications such as Facebook/WhatsApp and previous research. Although WeChat has become an integral part of everyday life for many users, research has only recently begun to examine the impact of this development on the societal and individual levels. The present review summarizes the literature on this topic with a focus on the motives to engage in using the app and potential detrimental effects of excessive use. In the context of the growing popularity and increasing usage times of the app – in particular in Asian countries – future research seems warranted to examine systematically how social media platforms such as WeChat will affect interpersonal communication behavior, well-being, and mental health. The direct comparison of WeChat’s influence on the mentioned variables compared with its competitors Facebook and WhatsApp often used in Western countries will also be of high importance.

## Background: What Is WeChat?

WeChat (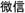, *Wēixìn*, micro-message) was first released by the Chinese multinational company Tencent Holding Limited in January 2011. With currently more than one billion monthly active users (Tencent, 2018), it has become one of the most important applications on smartphones in China. WeChat presents a multipurpose smartphone application, going beyond the features offered by its counterpart WhatsApp popular in Western countries. The multipurpose platform WeChat integrates a variety of services such as messaging, socialization, and mobile payment services, and steadily expands its functionality by integrating new services such as city services allowing users, e.g., to book transportation or to pay for traffic fines in China’s metropolitan areas. In the context of social media use, the core functions of WeChat include messaging services with other users and/or sharing photos/videos via the *moments* function. In line with the features of WhatsApp, its Chinese counterpart offers free video and voice call features and a large range of emoticons to emphasize the emotional state of the users.

It is of importance to mention that the social media part of this smartphone application represents only one relevant aspect of WeChat. Indeed, WeChat must be seen in broader terms than “just” being social media. From a different angle, WeChat can be seen as a platform to acquire various information. The function of *WeChat-Public-Account* enables users to get any kinds of information when they follow a distinct public account. In fact, increasingly organizations, e.g., enterprises, universities, and governments, have utilized a *WeChat-Public-Account* as a channel to transmit information to specific users and interact with them.

An important new feature was added to WeChat in January 2014, with the inclusion of the *red envelope* (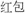, *hóngbāo*) function. This function mirrors the Chinese tradition to exchange monetary gifts via a red envelope (in particular during the Chinese Spring Festival). This red envelope function became immediately so successful that more than 768 million individuals participated in sending and receiving red envelopes during the six-day Spring Festival holiday in 2018 (Xinhua, 2018). Additional numbers on WeChat behavior have been reported recently underlining the importance of WeChat. In September 2017, 902 million individuals logged in daily to WeChat and 38 billion messages have been sent over this platform every day. The average user spent 139 mins each month on the app to call and 68 million videos have been posted each day in 2017 (all numbers taken from Blog.WeChat.com, 2017). These numbers reflect that WeChat has become an integral part of everyday life for many users.

Despite the increasing growth of WeChat, research on the potential effects of WeChat usage on the societal and individual levels is currently scarce. Several reasons may have contributed to the lack of systematic research in this field. First, despite growing research on the effects of social media use, previous research has mainly focused on the most popular social media platforms in Western countries, such as Facebook/WhatsApp [e.g., see a review on Facebook research by Wilson et al. (2012) and Montag et al. (2015b)]. However, given potential cultural differences as well as the more complex integrated functions of WeChat, the previous findings in the Western countries may not be simply extended to determine potential impacts of WeChat in China and other countries with high-usage numbers. Second, despite its popularity in Asia, WeChat is still not well known and used by many individuals living in Western countries. As switching costs are high, it is not easy for them to change from the current social media (e.g., Facebook) to a new one (e.g., WeChat).

**FIGURE 1:**
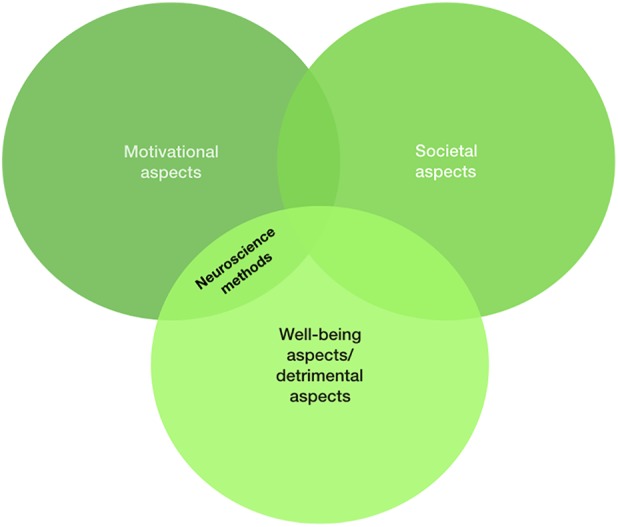
The present review focuses on motivational (section “Motivational Aspects of WeChat Usage”), well-being (together discussed with detrimental aspects in the section “Detrimental Aspects of WeChat Usage on Mental Health”), and societal aspects (section “WeChat Usage: A Societal Perspective”) in the context of WeChat usage. Note that research areas of “Motivational Aspects of WeChat Usage” and “Detrimental Aspects of WeChat Usage on Mental Health” are closely entwined and illuminating relations between these areas might in particular also be guided by neuroscientific research.

In the context of the steadily growing numbers of WeChat users and its rapid extension of functions over the past years, which led to highly interwoven interactions with everyday life, specific research on the effects on the societal and individual levels is needed. From our perspective, research needs to focus on different perspectives to account for the complex effects, which will be outlined after reviewing currently available empirical research on WeChat. In the present work we will focus on three different research approaches with high relevance for WeChat research (also illustrated in Figure 1). We will discuss motivational aspects of WeChat usage (see section “Motivational Aspects of WeChat Usage”) along with potential detrimental aspects of WeChat use on mental health (see section “Detrimental Aspects of WeChat Usage on Mental Health”). Clearly, these fields are entwined. Although motivational aspects first aim at understanding why individuals use WeChat, some of these motivational aspects might be associated with detrimental aspects. A review by Ryan et al. (2014) on Facebook addiction revealed that several factors of the uses and gratification theory such as searching for companionship when being lonely might be linked to addictive tendencies toward Facebook, but more in general “inconsistency in the measurement of Facebook addiction makes it difficult to propose compelling arguments regarding this condition” (p. 145). In short, being depressed and/or lonely might result in searching for and perhaps also finding online support. This positive reinforcement might further result in a transit from habitual to excessive usage of Facebook without solving one’s own social problems in the offline world. Moreover, lonely persons might be in particular prone to get depressed due to processes of social comparisons triggered by the many happy and exaggerated Facebook profiles demonstrating the often superficially perfect lives of other users (Song et al., 2014; Tandoc et al., 2015). The investigation of motivational/well-being/detrimental areas of social media usage – in the present context WeChat – might also strongly benefit from neuroscientific studies, because brain processes underlying these psychological processes are in part well understood and provide an additional layer of information explaining why individuals use social media platforms and/or develop addictive tendencies (Montag et al., 2017). For this reason, the present review also presents initial neuroscientific findings on WeChat usage/addiction. Finally, and as depicted in Figure 1, the present review also provides some first insights into the effects of WeChat usage on a societal level (see section “WeChat Usage: A Societal Perspective”) before concluding with a brief outlook on future research questions.

## Overview on Previous Empirical Work: Motivations That Drive WeChat Usage and Potential Detrimental Effects of Excessive Usage

### Motivational Aspects of WeChat Usage

Early work on WeChat usage aimed at understanding motivational aspects of WeChat usage. In this context, Gan and Wang (2015) (an interview study with 18 participants), and Wang et al. (2015) pointed out that the need for social exchange represents one of the strongest driving forces to use WeChat. This view can be supplemented by the findings of Mao (2014) showing in 200 students that WeChat is also used to relax and for stress relief. On one hand, the students of this study in particular appreciated that WeChat enables them to stay in contact with friends; however, and on the other hand, they also stated that their own peers encourage them to use WeChat more often [hence social pressure may be a driving force promoting excessive and potentially problematic use, see also the next section and the new work by Li et al. (2018) on stressful life events, WeChat addiction and life satisfaction]. Going beyond the work by Mao (2014), Lien and Cao (2014) provided evidence that motivational factors such as (satisfying the need for) entertainment, sociality, and information on WeChat impact the attitude toward WeChat. The attitude toward WeChat itself was linked to positive word of mouth (speaking positively about WeChat and its functions), whereas both the attitude toward WeChat and the variable of word of mouth were positively influenced by the factor trust in WeChat (see page 108 in Lien and Cao, 2014). Of note, Pang (2016) identified four factors predicting WeChat usage: passtime, affection, sociability, and fashion. Although the *frequency of WeChat use per week* was best predicted by the *passtime* variable (e.g., WeChat being pleasant rest, fun, and relaxant), *time spent on each session* was best predicted by the factor *affection* (e.g., help and thank other people).

Similar to the well-known “Like”-function on Facebook or Instagram, WeChat allows users to comment and like posted *moments* of their friends. Evidence from previous fMRI research on these social media platforms (Sherman et al., 2016, 2018) indicates that posted pictures getting many “Likes” compared with posted pictures getting fewer “Likes” results in stronger activity of the nucleus accumbens region/ventral striatum, hence reflecting the activation of reward-related mechanisms in the human brain (Sherman et al., 2016). These effects might be more pronounced in adolescent users (compared to older adults), possibly due to the lower top–down regulation in the adolescent prefrontal cortex undergoing maturational processes. This idea still needs to be more investigated: Sherman et al. (2018) “only” contrasted young age groups with (even) positive age associations and accumbens activity in the context of processing popular pictures with high vs. few “Likes” in High School students. Future studies will also need to contrast younger with much older participants. For a more in-depth overview see also Montag (2018). It can be expected that comparable rewarding mechanisms (self-related positive feedback) as observed for receiving a “Like” on Facebook/Instagram may also underlie the appeal of sharing pictures on WeChat. Adding to this, a recent work by Gan (2017) observed that the following motives (in a descending order) were of importance to predict giving a “Like” for a posted *moment* on WeChat: enjoyment (hedonic gratification), providing support for another person (social gratification) and searching for information on WeChat (utilitarian gratification). In line with the rewarding aspects of receiving “Likes” (as apparent from research on Facebook and Instagram), social aspects contribute to the rewarding effects such as it has been reported that receiving “Likes” is self-assuring and increases self-esteem of the user (Nie et al., 2018). Although some aspects of psychological/neuroscientific research on the Western platforms such as Facebook may generalize to WeChat usage (e.g., both the platforms use the “Like”-mechanism to attract and prolong usage times), findings may not always simply extend to WeChat. This is certainly due to the many different features available on the respective platforms. In Table 1 we provide an overview on the available features of the prominent platforms WeChat, Facebook, and WhatsApp. Going beyond this, cultural differences between the users hamper a simple generalization of findings, e.g., with respect to motivational aspects, in particular since Facebook and WhatsApp are mostly used in Western countries, whereas WeChat clearly is, to a great extent, a Chinese/Asian phenomenon.

**Table 1:** Overview on prominent (not all) functions available or not available in the applications of WeChat, WhatsApp, and Facebook (note that we compare the Facebook app and not the Facebook messenger, here^∗^).

	WeChat	WhatsApp	Facebook
Texting peer to peer(s)	Yes	Yes	Yes
Video and picture posting functions via peer to peer(s)	Yes	Yes	Yes
Group chatting	Yes	Yes	Yes
Like mechanism (liking a post from another person)	Yes	No	Yes
Newsfeed	Yes	No	Yes
Payment option	Yes	No	No
Red envelope	Yes	No	No
Public account in application	Yes	No	Yes
City services	Yes	No	Yes
Mobile games	Yes	No	Yes
Availability via mobile app only	Yes^∗∗^	Yes^∗∗^	No

*^∗^Complicating things, WhatsApp belongs to Facebook. Please note that this overview represents the current state of affairs when this review was written. New features might be added, soon. ^∗∗^Desktop versions run only via initial activation over one’s own smartphone.*

Aside from these first insights into motivational aspects of general WeChat usage, some works attempted to explore factors that drive WeChat users’ continuance intention of the app. Applying the theoretical framework of uses and gratification (e.g., Ruggiero, 2000), Gan and Li (2018) demonstrated that *media appeal* is by far of highest importance to predict the *intention to continue WeChat usage*. Individuals scoring high on the psychological construct *media appeal* are among others known to like WeChat because it enables them to communicate with others immediately and to do this via an easy and cost-effective way. Gan (2016) also revealed that gratifications of entertainment, information, and reward significantly affect WeChat users’ intention to follow the above-mentioned public accounts. In this context also a work by Zhang et al. (2017) is noteworthy, providing support that the factors “social value and hedonic value influence continuance intention” of WeChat (p. 284). In the same realm, Lien et al. (2017) among others demonstrated the relevance of the user’s satisfaction with WeChat to predict continuance intention. Meanwhile, effects of Chinese cultural factors have been paid attention to in recent years. Through introduced guanxi-based constructs into the technology acceptance model, Chen et al. (2017) found that gǎnqíng (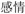; see detailed explanation further below in section “Final Conclusion and Outlook on Important Research Perspectives”) positively affects continuance intention to use WeChat, whereas mìanzǐ (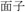, “face”) has a negative impact. For more completeness, we also point to the work by Zhang et al. (2016) investigating why persons in general discontinue with their social media use.

### Detrimental Aspects of WeChat Usage on Mental Health

A second area of research in the realm of WeChat usage focused on potential detrimental effects of using this application in excessive amounts. A work by Wen et al. (2016) demonstrated that WeChat usage itself is neither good or bad *per se*, but that the kind of usage matters. Using WeChat to follow own interests is even associated with higher life satisfaction. In contrast, there have been a growing number of studies showing that smartphone use disorder/smartphone addiction (SA)^1^ might be associated with poorer sleep quality, because these users stick around their smartphones too long, also in the evening or at night (Van den Bulck, 2003; Demirci et al., 2015). Interestingly, a new study by Xu et al. (2016) observed that WeChat users have *better* sleep quality compared with non-users. The authors explained the initial contradictory findings in the context of stress relieving properties of WeChat [see also above the work by Mao (2014)]. Nevertheless, the study by Xu et al. (2016) did not assess problematic or potentially addictive use tendencies in the WeChat users, which might be specifically associated with negative emotions [see link between negative emotions and Internet Use Disorder/Internet addiction (IA) in Montag et al., 2016b] and negative sleep quality (e.g., Demirci et al., 2015; Lemola et al., 2015).

Recent research suggests that excessive WeChat use may resemble core components of addictive behavior: Montag et al. (2018) provided a new questionnaire to assess “WeChat addiction” (WCA) including the symptoms of loss of control over usage and social problems due to usage that in part resembles symptoms that characterize addictive disorders such as substance-use disorders or behavioral addictions such as pathological gambling (see, e.g., also diagnostic criteria for substance use disorders and behavioral addictions in DSM-5 or ICD-11). Higher scores on this scale thus reflect more problematic and possibly addictive use. Integrating additional assessment of MRI-based measures of brain structure, this study demonstrated that higher levels of WCA symptoms were associated with lower gray matter volumes of the subgenual anterior cingulate cortex, a key region for regulatory control and emotional conflict adaptation. These findings complement the previous findings by Zhou and Wang (2017) demonstrating associations between higher scores on WCA and lower self-control, and the findings by Hou et al. (2017) associating higher WCA tendencies with a higher external locus of control. Individuals with a higher external locus of control believe that their lives are more driven by external factors such as fate or destiny. An empirical survey of 1365 Chinese adolescents showed that high life stress and external locus of control result in increasing risk for behavioral problems (Liu et al., 2000). Finally, a recent work by Hou et al. (2018) also demonstrated associations between higher WCA and the personality trait of neuroticism, a general risk factor for psychopathology, particularly internalizing disorders including depression and anxiety (Lahey, 2009). In addition, this user group additionally exhibited lower agreeableness scores, a personality trait closely linked to substance use disorders as pointed out in a meta-analysis by Kotov et al. (2010). Additional personality traits have also been shown to account for individual differences in WeChat usage, such as posting more selfies on WeChat has been associated with higher extraversion (Guo et al., 2018). Wang (2017) pointed out that this kind of self-presenting/promoting behavior might additionally be associated with the personality trait of narcissism. Finally, Lin and Lei (2016) observed that the personality of a WeChat user could also predict if he or she receives many “Likes” on the shared *moments*. In sum, personality represents an important construct to understand WeChat usage. For an introduction into personality see Montag and Panksepp (2017) and in detail for the Five-Factor-Model of Personality the work by McCrae and John (1992).

### WeChat Usage: A Societal Perspective

A third area in WeChat research initially explored the impact of WeChat usage on the societal level. Western researchers are increasingly concerned about the impact of filter bubbles and echo chambers due to personalized news feeds such as on Facebook undermining democratic processes (Zuiderveen Borgesius et al., 2016; see also the potential and problems of psychological profiling/microtargeting; Kosinski et al., 2015; Matz and Netzer, 2017). Among others, informing yourself via a personalized news feed, which is presented to you according to your “Like” profile, might lead to biased views on the world and even radicalization, in particular if persons inform themselves only via social media about the daily news [see, for an introduction of the term filter bubble, the work by Pariser (2011)]. Please see also Table 1 showing that both Facebook and WeChat have such a news feed included in their application informing users on their social networks but also aspects in line with their personalized interests. Note that effects of such a personalized news feed on pluralistic opinions in society might differ depending on the different forms of governance across global societies.

WeChat has reached an enormous popularity with great potential in the discourse of societal areas of relevance (e.g., DeLuca et al., 2016) and also using WeChat for educational purposes (Zeng et al., 2016). Given that also older generations are using WeChat in the meanwhile [Huang and Zhang, 2017; see also an additional small study on socio-demographics of WeChat usage by Deng and Lin (2015)], the study of the WeChat platform will be of interest to get insights into a wide array of human behavior. This might be of special relevance when considering the new *social credit system* of the Chinese government used as a tool to develop a national reputation system of the Chinese population (BBC.com, 2015).

## Final Conclusion and Outlook on Important Research Perspectives

Most of the recent research unfortunately only relies on self-report techniques, but clearly it will be of relevance to do digital phenotyping via the methods of *Psychoinformatics* to get exact information on what people are doing on WeChat (Montag et al., 2016a; Insel, 2017). A more objective and quantitative assessment of app usage is not only of relevance to assess problematic behavior of smartphone/WeChat use behavior that is often biased in self-reports (Lin et al., 2015; Montag et al., 2015a, 2016a), but might be used in the context of mental health applications, e.g., to determine patterns of use that may predict depression [such as recently observed by Elhai et al. (2017) in Western populations]. Clearly, this raises many problems in the area of data security in need to be solved in the near future to be able to use data from *Psychoinformatics* in the health sector (Markowetz et al., 2014).

Aside from this, it will be necessary to take a detailed look at the many possible distinct areas of WeChat behavior clearly going beyond broad WeChat usage. The necessity to focus on the actual behavior on a platform has also been stressed in a recent work by Rothen et al. (2018) investigating problematic Facebook use. Note that in the work on WeChat by Montag et al. (2018) it also has been shown that frequency of paying was inversely linked to the gray matter volume of the nucleus accumbens, a key reward-processing region of the brain. Therefore, self-report studies and also studies employing methods from *Psychoinformatics* need to assess the specific functions used and how often such distinct behaviors are shown. Again, we point toward the manifold features of WeChat indeed making it a multipurpose platform. In sum, it is not only of relevance to understand how long or frequent a person is using WeChat on a daily level, but clearly what kinds of activities are shown on an individual level and whether this leads to problems in everyday life.

Going beyond the already mentioned points, a good understanding of WeChat usage in Chinese/Asian participants will only be possible if psychological constructs will be investigated being closer linked to Chinese culture. Among these are concepts such as guānxì (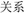, Chen et al., 2017), or personality dimensions going beyond the famous Big Five (Cheung et al., 1996, 2001). Guānxì, as a unique feature of Chinese society, consists of different aspects such as gǎnqíng (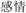, affection), rénqíng (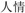, favor), and xìnrèn (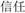, trust) (Jukka et al., 2017). Recent studies have paid attention to the roles of different guānxì (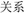) dimensions exerting effects on the usage of social media (Chen et al., 2017; Davison et al., 2018). Much attention also should be paid to factors impacting different WeChat usage behavior, such as discontinuance behavior of WeChat and switching behavior to other social media (e.g., Turel, 2015, 2016; York and Turcotte, 2015). In fact, the increasing functions and more popularity of platforms such as Facebook and WeChat (e.g., see Table 1) have already resulted in some negative phenomena, such as technology/system overload (Karr-Wisniewski and Lu, 2010), information overload (Jacoby et al., 1974), and social overload (Maier et al., 2015a); and went along with negative emotional and behavioral outcomes, such as stress, conflict, and discontinuance (Maier et al., 2015b; Turel, 2015, 2016).

Future work will also need to determine further effects of excessive and problematic use on the brain and whether alterations associated with problematic use mirror neural changes that have been established in addictive disorders. Moreover, the initial findings by Montag et al. (2018) that point to potential brain structural changes associated with excessive use of WeChat were observed in a cross-sectional design, thus it remains to be determined whether the observed changes represent predisposing alterations for escalating usage or consequences of excessive use. In short, longitudinal studies are of high importance [such as the structural MRI study by Zhou et al. (2017) investigating the effects of gaming on the human brain], to disentangle the predisposing factors from the consequences of digital use.

In the realm of the recently introduced concept of *Internet Communication Disorder* (Wegmann and Brand, 2016; Montag et al., 2018), we have already mentioned above that lower gray matter volumes of the subgenual anterior cingulate cortex have been associated with higher WCA. Indeed, it is not clear, if *Internet Communication Disorder* and WCA, or perhaps better called *WeChat Use Disorder* (again see footnote 1 describing problems in terminology), might warrant an own disorder in a future edition of the *International Classification of Diseases* (ICD) issued by the World Health Organization (WHO). Nevertheless, the recent inclusion of *Gaming Disorder* in ICD-11 underlines the need to study behavioral addictions, as also recently emphasized by a current commentary by Potenza et al. (2018) in *Nature*. Moreover, important psychological questions to be answered in the context of WeChat usage will also touch the area of a *digital etiquette* (Montag and Diefenbach, 2018), and more detailed, if WeChat usage leads to lower social connection/taking less care of one’s own children (Kushlev and Dunn, 2018), possibly also resulting in lower empathy (Melchers et al., 2015; Lachmann et al., 2018). A new study by Kushlev et al. (2019) also demonstrated that smartphone usage reduces smiles between strangers. It also needs to be better understood at what levels WeChat may benefit users in everyday life and may even enhance resilience due to its potential stress-relieving effects or increased social support. This might be possible in many areas including administration of everyday life issues, health, and obviously communication.

In sum, the present article demonstrated that WeChat represents a multipurpose platform including social media functions, but clearly also going beyond this (see Table 1). Researchers interested in cross-cultural research on social media usage will need to consider this, because this fact complicates matters when one aims to understand if same mechanisms globally can explain why humans are attracted to social media applications. Studies directly comparing individuals using WeChat, Facebook, and WhatsApp (but also other platforms such as Twitter or Weibo) with respect to motivation to use the app and potential effects on well-being are needed to address common and distinct features. Unfortunately, this task will only be hard to achieve given that some platforms are not available in all countries and certain platforms are dominating domestic markets with low chances for competitors to get a huge share of an already saturated market.

## Author Contributions

CM drafted the first version of the review. BB and CG critically worked over this draft and revised it before submission.

## Conflict of Interest Statement

The authors declare that the research was conducted in the absence of any commercial or financial relationships that could be construed as a potential conflict of interest.

## Footnotes

1 Note that SA is not an official diagnosis yet. For an introduction see Duke and Montag (2017); moreover, see that a specific from of Internet addiction, namely Gaming Disorder, has been included in the beta-draft of the ICD-11 as a distinct disorder (ICD.who.int, 2018). We also note that the introduced concept of WeChat addiction is not officially recognized and it is unclear at the moment if excessive usage of a platform such as WeChat is best characterized by the term addiction/disorder or else. Clearly, we aim at not to overpathologize everyday life (see Billieux et al., 2015).
